# Patient costs of hypertension care in public health care facilities in Kenya

**DOI:** 10.1002/hpm.2752

**Published:** 2019-02-14

**Authors:** Robinson Oyando, Martin Njoroge, Peter Nguhiu, Fredrick Kirui, Jane Mbui, Antipa Sigilai, Zipporah Bukania, Andrew Obala, Kenneth Munge, Anthony Etyang, Edwine Barasa

**Affiliations:** ^1^ Health Economics Research Unit KEMRI‐Wellcome Trust Research Programme Nairobi Kenya; ^2^ Clinical Unit, KEMRI Centre for Clinical Research Nairobi Kenya; ^3^ Epidemiology and Demography, KEMRI Centre for Geographic Medicine Research, Coast Kilifi Kenya; ^4^ Public health nutrition, maternal and child health unit, KEMRI Centre for Public Health Research Nairobi Kenya; ^5^ Medical Microbiology and Parasitology, Moi University Eldoret Kenya; ^6^ Nuffield Department of Medicine University of Oxford Oxford UK

**Keywords:** catastrophic costs, hypertension, Kenya

## Abstract

**Background:**

Hypertension in low‐ and middle‐income countries, including Kenya, is of economic importance due to its increasing prevalence and its potential to present an economic burden to households. In this study, we examined the patient costs associated with obtaining care for hypertension in public health care facilities in Kenya.

**Methods:**

We conducted a cross‐sectional study among adult respondents above 18 years of age, with at least 6 months of treatment in two counties. A total of 212 patients seeking hypertension care at five public facilities were interviewed, and information on care seeking and the associated costs was obtained. We computed both annual direct and indirect costs borne by these patients.

**Results:**

Overall, the mean annual direct cost to patients was US$ 304.8 (95% CI, 235.7‐374.0). Medicines (mean annual cost, US$ 168.9; 95% CI, 132.5‐205.4), transport (mean annual cost, US$ 126.7; 95% CI, 77.6‐175.9), and user charges (mean annual cost, US$ 57.7; 95% CI, 43.7‐71.6) were the highest direct cost categories. Overall mean annual indirect cost was US$ 171.7 (95% CI, 152.8‐190.5). The incidence of catastrophic health care costs was 43.3% (95% CI, 36.8‐50.2) and increased to 59.0% (95% CI, 52.2‐65.4) when transport costs were included.

**Conclusions:**

Hypertensive patients incur substantial direct and indirect costs. High rates of catastrophic costs illustrate the urgency of improving financial risk protection for these patients and strengthening primary care to ensure affordability of hypertension care.

## 1 BACKGROUND

The goal of universal health coverage (UHC) is to ensure that everyone has access to quality health care services they need without the risk of financial hardship.^1^, ^2^ Financial protection against the cost of illness is therefore a key policy objective of UHC. 3 The double burden of communicable and noncommunicable diseases (NCDs) in many low‐ and middle‐income countries (LMICs) places an additional burden on the already constrained health resources and is a threat to the attainment of UHC goals.^4^, ^5^, ^6^, ^7^

Kenya is committed towards attaining UHC by 2022.^8^ The Kenyan health sector is funded by different sources^9^ including; the government, out‐of‐pocket payments (OOP) and donors.^10^, ^11^, ^12^, ^13^, ^14^ The high level of OOP payments increases the risk of catastrophic health expenditure where households spend a large proportion of their budget on health care, which consequently have negative implications on living standards as they forego other goods and services.^15^ The incidence of catastrophic health care expenditure (at the 40% of annual non‐food expenditure threshold) related to direct health care payments to providers in Kenya is estimated to be 4.52%.^16^ In addition, it has been shown that households with a member with an NCD are two times more likely to incur catastrophic health expenditure compared with households where no member has an NCD.^16^ A minority of individuals (19%) in Kenya have health insurance coverage,^17^ and almost all employees in the formal sector, which is less than one‐fifth of those employed, are covered through the National Hospital Insurance Fund (NHIF).^18^, ^19^

Several studies done in Kenya have identified a high prevalence of hypertension (ranging from 12.3% to 50.1%).^6^, ^20^, ^21^ Examining the costs incurred by patients to access hypertension care is necessary as evidence suggests they are a barrier to access.^22^, ^23^ Knowledge of such costs is limited in Kenya. The objective of this study was to determine the costs associated with accessing hypertension care from the patient perspective.

## 2 METHODS

### 2.1 Study setting

The study was conducted from June to December 2017 in two sites in Kenya (Kilifi and Bungoma counties) purposively selected to reflect a diverse set of demographic, socio‐economic, and geographical settings. Kilifi is located on the coast of Kenya, and a high burden of stroke and heart failure has been described in this area.^24^ The population in Kilifi has been well characterized by data from the health and demographic surveillance system run by the KEMRI Wellcome Trust Research Programme.^25^ The Webuye Health and Demographic Surveillance System run by Moi University is located in Bungoma County in the western region of Kenya.^26^ Multiple cardiovascular risk factors have been identified in this area.^27^ This was part of a larger study that assessed the health system performance and the capacity for effective coverage of diabetes mellitus and hypertension services at primary care in the context of a devolved health system (KEMRI Scientific Ethics and Review Unit protocol number 3270).

Six public health care facilities were purposively selected in consultation with county health officials to generate a sample of facilities with different workloads, plus the location of the clinics relative to the communities served. However, due to the nationwide nurses' strike at the time of data collection,^28^ data were collected from five facilities unlike the anticipated six facilities in the two counties. In Kilifi, a public hospital and a health centre that provided hypertension treatment were selected while in Webuye, three public hospitals were sampled. For this descriptive analysis, data from all the facilities were pooled.

### 2.2 Sample size and sampling

The target enrolment was 278 patients for a sample size sufficient to obtain a precise estimate of hypertension patient costs based on the formulae by Kirkwood^29^:

$$N={\left(Z_{\alpha /2}+Z_{\beta }\right)}^{2*}\left(P\;\left(1–P\right)\right)/e^{2}),$$

where

Z_α/2_: is the critical value of the normal distribution at α/2 (for a confidence level of 95%, α is 0.05 and the critical value is 1.96)
Z_β_: is the critical value of the normal distribution at β (for a power of 80%, β is 0.2 and the critical value is 0.84)
P: is the expected true proportion of hypertension in the population in Kenya of 15% (0.15)
e: is the desired standard size of standard error around the estimated proportion of 6% (+/− 0.06).

Every hypertensive patient receiving treatment and available at participating facilities during data collection was approached to participate in this study. Patients were eligible if they self‐reported hypertension diagnosis, had received treatment for a minimum of 6 months after diagnosis, and were more than 18 years of age. Consenting patients were selected based on meeting the eligibility criteria and the order of arrival at the clinic. Respondents were asked to report on their health service use, associated costs, income, and coping mechanisms if they undertook any of the following to meet hypertension care costs: borrowing (having taken a loan), selling household items or assets (eg, livestock), and use of savings.

### 2.3 Measuring patient costs

Interviews were conducted using a structured questionnaire. Three trained interviewers collected the data. Interviews were conducted primarily in Kiswahili, with local languages (Giriama and Bukusu in Kilifi and Bungoma, respectively) used to clarify questions where necessary. Respondents were asked about costs incurred for different care seeking episodes described in Table 1.

**Table 1:** Care seeking episodes included in patient cost analysis

Care Seeking Episode	Description	Recall Period
Sick visit	Cost of current care seeking and any out‐patient visit when the patient fell ill due to hypertension outside the scheduled clinic appointments	1 mo
Inpatient visit	Cost of admission due to hypertension	12 mo
Drug collection visit	Cost of regular medication prescribed to the patient to manage hypertension	Frequency of drug collection, ie, monthly/quarterly
Laboratory/diagnostic visits	Cost of routine lab/diagnostic services done at a health facility	Frequency of lab/diagnostic services, ie, monthly/quarterly
Scheduled clinic check‐up visits	Costs due to regular clinic appointments	Frequency of clinic appointments, ie, monthly/quarterly

To annuitize sick visit costs, we summed up costs incurred during current care visit and any reported outpatient visit costs that occurred due to hypertension in the last 4 weeks and then multiplied by 13 (assuming there are 52 weeks in a year). On the other hand, to annuitize costs in other care seeking episodes described in Table 1, reported costs were multiplied by the number of visits, ie, weekly, monthly, or quarterly for each episode. Furthermore, any inpatient admission costs in the last 1 year was also collected. Overall hypertension care costs for all care seeking episodes were calculated by summing up the annual costs in each care seeking episode.

For each of the care seeking episode, two broad costs categories were estimated: direct OOP costs and indirect (productivity losses) costs for both patients and their caregivers. Direct health care costs included any charges levied for medicines and user fees, ie, registration, consultation, and laboratory services. Direct non–health care costs included transport costs to and from a health provider and any costs associated with food and accommodation while seeking care. For this descriptive analysis, OOP costs were defined as the sum of direct health care and direct non–health care costs. Analysis was restricted to patients who reported any OOP costs for each care seeking episode.

Indirect costs were estimated based on the total hours lost while seeking care as well as the cost of illness‐related to lost home productivity for both patients and their caregivers, assuming that these hours would have been used for productive activity in the absence of hypertension.^30^ Income lost due to hypertension illness was therefore estimated by multiplying the estimated number of lost production hours due to hypertension by the official minimum wage (US$ 84 per month) in the agricultural sector in 2017 given the main economic activities in our study sites.^31^ We assumed an average workday of 8 hours per day and 22 working days per month. Caregivers' lost home productivity was also estimated by multiplying the total number of hours spent by caregivers caring for the patient by the official minimum wage rate.

Income was estimated by asking detailed questions about income categories, including patient income, income for household members, welfare payments, and government assistance. As a measure of financial risk protection, we compared total direct costs incurred against annual household income and total direct costs excluding transport costs and defined costs as catastrophic if they exceeded 10% of household income.^32^

### 2.4 Data management and analysis

Data were double‐entered by two different individuals to enhance data quality. The two data sets were compared to eliminate data entry errors. Consistency and range checks were used to ensure completeness of data. Data were analysed using STATA 14.0 (STATA, Statacorp, Texas). Frequency counts and percentages were used to describe patient demographic and socio‐economic variables. Due to skewed nature of costs data, mean and median values were reported for all cost estimates as a measure of central tendency, and 95% confidence intervals (CI) and interquartile range (IQR) were reported. Cost information (in Kenya shillings was converted to United States Dollars [US$] using the following exchange rate: US$ 1 = KES 102 (average exchange rate, January to December 2017; Currency Converter/Foreign Exchange Rates/OANDA. http://www.oanda.com/currency/converter/).

## 3 RESULTS

### 3.1 Participants characteristics and health services utilisation

In total, 212 individuals were interviewed, 114 (54%) from Kilifi. Less than half of the sampled patients had secondary level education, were formally employed, or were subscribed to a health insurance scheme (Table 2). More than 80% of the patients were diagnosed with hypertension at a public facility and had been on treatment for at least 1 year (Table 2). The median travel time to a health facility was 30 minutes (IQR, 19‐60). Ninety‐one percent (95% CI, 86.3.2‐94.2) of the respondents used public means of transport to get to the health facility while 9.0% (95% CI, 5.8‐13.7) walked. Fifty‐eight patients reported that the facility they attended was not the nearest to them; of these, 43.1% (95% CI, 30.7‐56.4) reported referral, 41.4% (95% CI, 29.2‐54.7) reported lack of medicines and/or diagnostic facilities, and 15.5% (95% CI, 8.1‐27.6) reported other reasons for not visiting the nearest health facility.

**Table 2:** Patient characteristics

Characteristic	(n) Observations	Proportion (95% CI)
Mean age in years	212	60 (58.2‐61.8)
Gender		
Male	(212) 50	23.6% (18.3‐29.8)
Female	(212) 162	76.4% (70.2‐81.7)
Highest education level		
None	(212) 61	28.8% (23.0‐35.3)
Primary	(212) 82	38.7% (32.3‐45.5)
Secondary	(212) 53	25.0% (19.6‐31.3)
Higher	(212) 16	7.5% (4.7‐12.0)
Enrolled to a health insurance scheme		
Yes	(212) 46	21.7% (16.6‐27.8)
No	(212) 166	78.3% (72.2‐83.4)
Employment status		
Formal employment	(211) 42	19.9% (15.0‐25.9)
Informal/unemployed	(211) 169	80.1% (74.1‐85.0)
Breadwinner		
Patient	(211) 94	44.5% (37.9‐51.4)
Not patient	(211) 117	55.5% (48.6‐62.1)
Reason for not working		
Related to hypertension	(208) 46	22.1% (17.0‐28.3)
Not related to hypertension	(208) 162	77.9% (71.7‐83.0)
Where diagnosed		
Public facility	(212) 186	87.7% (82.5‐91.5)
Private facility	(212) 26	12.3% (8.5‐17.5)
Illness duration		
6 mo‐1 y	(212) 33	15.6% (11.3‐21.1)
1‐5 y	(212) 101	47.6% (40.9‐54.4)
>5 y	(212) 78	36.8% (30.5‐43.5)

### 3.2 Patient costs associated with health care use

#### 3.2.1 Sick visit costs

Thirty‐six percent of the patients sought care outside of their scheduled clinic appointments. Transport costs during a sick visit represented the highest cost category (42.2%) of all direct OOP costs. Patients incurred substantial indirect costs (mean annual cost, US$ 113.5; 95% CI, 92.6‐134.3) for such outpatient sick visits (Table 3).

**Table 3:** Mean and median annual hypertension care cost at five public facilities in Kenya (2017 US$)

Care Seeking Episode	Cost Category	Observations	Mean US$ (95% CI)	Median US$ (IQR)	As a % of Total Direct Costs
Sick visit	Direct health care costs				
	User charges	76	79.0 (49.2‐108.8)	31.2 (12.7‐71.4)	18.2
	Medicines	76	140.8 (91.7‐189.9)	63.7 (17.5‐175.9)	38.3
	Direct non–health care costs				
	Transport	76	173.9 (58.0‐289.7)	70.1 (25.5‐152.9)	42.2
	Food	76	4.6 (0.9‐8.2)	0	1.3
	Subtotal (direct costs)	76	398.2 (242.7‐553.7)	214.8 (89.9‐470.3)	
	Indirect costs	76	113.5 (92.6‐134.3)	87.1 (55.4‐136.9)	
	Direct + indirect costs	76	511.7 (348.2‐675.2)	327.9 (143.2‐624.3)	
In‐patient admission	Direct health care costs				
	User charges	23	50.8 (25.1‐76.5)	32.1 (9.8‐63.7)	49.8
	Medicines	22	24.0 (12.3‐35.6)	16.6 (3.4‐34.3)	22.5
	Direct non–health care costs				
	Transport	26	14.1 (5.4‐22.9)	5.9 (3.1‐15.7)	15.7
	Food^a^	10	28.3 (3.3‐53.3)	10.3 (7.8‐54.9)	12.0
	Subtotal (direct costs)	26	90.3 (48.7‐131.8)	64.5 (19.8‐102.0)	
	Indirect costs	26	67.2 (37.3‐97.1)	31.2 (16.3‐108.7)	
	Direct + indirect costs	26	157.5 (99.1‐215.8)	124.9 (46.3‐231.6)	
Medicine collection	Direct health care costs				
	Medicines	152	37.4 (29.0‐45.8)	20.6 (7.8‐45.9)	75.2
	Direct non–health care costs				
	Transport	43	36.4 (25.5‐47.3)	23.5 (8.2‐70.6)	20.7
	Food	13	24.0 (7.3‐40.6)	11.8 (8.8‐23.5)	4.1
	Subtotal (direct costs)	163	46.4 (36.8‐55.9)	23.5 (9.4‐58.8)	
	Indirect costs	212	38.8 (33.6‐44.0)	29.1 (14.8‐51.0)	
	Direct + indirect costs	212	70.6 (60.3‐81.0)	45.0 (23.0‐83.6)	
Diagnostic visit	Direct health care costs				
	Test	30	9.3 (6.0‐12.7)	6.5 (2.4‐11.8)	14.4
	Direct non–health care costs				
	Transport	38	36.1 (25.9‐49.9)	23.5 (11.8‐47.1)	74.2
	Food	9	24.4 (11.5‐37.3)	23.5 (11.8‐35.3)	11.3
	Subtotal (direct costs)	61	31.8 (21.9‐41.7)	14.1 (5.9‐47.1)	
	Indirect costs	202	33.2 (28.3‐38.0)	23.4 (11.5‐40.2)	
	Direct + indirect costs	202	42.6 (35.5‐50.0)	28.7 (11.5‐43.1)	
Scheduled clinics	Direct health care costs				
	User charges	184	11.0 (9.0‐13.1)	6.7 (3.1‐11.8)	17.9
	Medicines	145	35.9 (26.4‐45.5)	16.5(5.9‐38.4)	45.8
	Direct non–health care costs				
	Transport	193	20.0 (16.0‐24.0)	11.8 (7.1‐23.5)	34.0
	Food	14	19.1 (8.3‐30.0)	11.8 (3.5‐35.3)	2.3
	Subtotal (direct costs)	212	53.6 (43.3‐63.9)	31.0 (14.1‐61.2)	
	Indirect costs	212	31.3 (26.2‐36.5)	23.0 (11.5‐37.4)	
	Direct + indirect costs	212	84.9 (71.4‐98.5)	54.6 (31.7‐91.7)	
Overall costs	Direct health care costs				
	User charges	187	57.7 (43.7‐71.6)	28.3 (16.7‐55.9)	16.7
	Medicines	162	168.9 (132.5‐205.4)	84.3 (26.3‐210.5)	42.4
	Direct non–health care costs				
	Transport	196	126.7 (77.6‐175.9)	58.8 (24.5‐128.4)	38.4
	Food	31	46.3 (28.3‐64.4)	27.0 (8.9‐72.5)	2.2
	Subtotal (direct costs)	212	304.8 (235.7‐374.0)	141.3 (70.0‐327.0)	
	Indirect costs	212	171.7 (152.8‐190.5)	141.4 (80.1‐209.9)	
	Direct + indirect costs	212	476.5 (397.8‐555.2)	282.7 (183.2‐552.9)	

Abbreviations: CI, confidence interval; IQR, interquartile range.

a Data include accommodation costs.

#### 3.2.2 Inpatient costs

The median number of hypertension‐related admissions the year preceding data collection was one with each inpatient admission lasting a median of four days (IQR, 2‐8). Of the total direct costs, user charges—especially during an inpatient admission—were a substantial cost compared with other care seeking episodes, with respondents spending an average of US$ 50.8 per annum. This was followed by food and/or accommodation costs (mean annual cost, US$ 28.3; 95% CI, 3.3‐53.3) (Table 3). Not surprisingly, indirect costs (mean annual cost, US$ 67.2; 95% CI, 37.3‐97.1) for an inpatient admission were high compared with direct costs.

#### 3.2.3 Medicine collection costs

**Figure 1:**
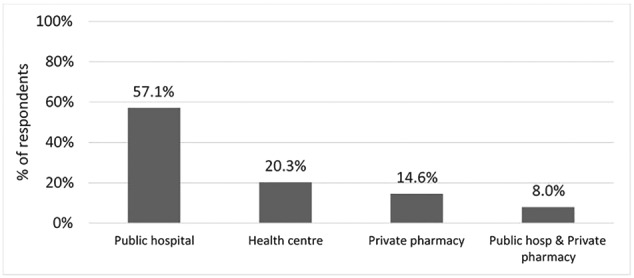
Source of hypertension medicines

Most patients incurred costs on monotherapy or two‐drug regimens at the time of interview (Table 4). Enalapril and hydrochlorothiazide was the most expensive combination of all prescribed antihypertensive medicines attracting mean annual cost of US$ 43.7 (95% CI, 29.2‐58.3) (Table 4). On the other hand, slightly more than half (57.1%) of the patients reported obtaining their routine medicines from a public hospital (Figure 1). Medicines accounted for 75.2% of total OOP costs during a medicine collection visit while transport and food accounted for 20.7% and 4.1%, respectively (Table 3).

**Table 4:** Drug combination costs

Drug Name	Observation	Mean US$ (95% CI)	Median US$ (IQR)
Enalapril	11	37.6 (18.8‐56.3)	47.1 (10.6‐56.5)
Hydrochlorothiazide	19	25.9 (14.2‐37.6)	21.2 (4.7‐47.1)
Nifedipine	51	31.7 (20.0‐43.5)	13.5 (3.9‐52.9)
Enalapril + hydrochlorothiazide	24	43.7 (29.2‐58.3)	40.6 (18.2‐61.8)
Nifedipine + enalapril	10	16.2 (6.3‐26.0)	10.6 (3.1‐22.7)
Nifedipine + hydrochlorothiazide	47	28.8 (17.6‐39.9)	17.6 (7.8‐33.5)
Nifedipine + hydrochlorothiazide + enalapril	10	36.6 (5.3‐68.0)	16.8 (7.1‐42.4)
Other combinations*	10	29.5 (15.7‐43.4)	30.6 (12.9‐38.2)

Abbreviations: CI, confidence interval; IQR, interquartile range.

Asterisk is used to describe “Other combinations” as indicated: * Aminosaliysilic Acid + Atorvastatin = 1; Aminosaliysilic Acid + Losartan = 1; Amlodipine + Aminosaliysilic Acid + Digoxin = 1; Methyldopa = 1; Amlodipine + Amitriptyline = 1; Atenolol= 1; Losartan + Atenolol = 1; Losartan = 1; Unknown = 2

#### 3.2.4 Diagnostic/laboratory test costs

The main routine diagnostic test for hypertensive patients was blood pressure and weight (mean annual cost, US$ 1.2; 95% CI, 0.3‐2.0). Diagnostic costs were likely low given that blood pressure and weight are checked for free at public health care facilities. Other reported tests were blood pressure (annual mean cost, US$ 1.3; 95% CI, 0.4‐2.2) and urinalysis and echocardiogram (annual mean cost, US$ 4.5; 95% CI, 1.8‐10.7). The mean annual direct cost for seeking diagnostic or laboratory test services was US$ 31.8 (95% CI, 21.9‐41.7) with transport costs accounting for 74.2% of total OOP costs (Table 3).

#### 3.2.5 Scheduled clinic appointment costs

Half (52.8%) of the respondents attended their routine clinics monthly. The highest direct cost category during scheduled clinic appointments was medicines accounting for 45.8% of total OOP costs followed by transport (34%), user charges (17.9%), and food (2.3%) (Table 3).

#### 3.2.6 Overall hypertension care costs

Overall, the average direct annual costs for all hypertension care seeking episodes was US$ 304.8 (95% CI, 235.7‐374.0). Under direct costs, medicines (mean annual cost, US$ 168.9; 95% CI, 132.5‐205.4) attracted the largest share of costs (42.4%) followed by transport (mean annual cost, US$ 126.7; 95% CI, 77.6‐175.9), which represented 38.4% of total patient costs. User charges (mean annual cost, US$ 57.7; 95% CI, 43.7‐71.6), food (mean annual cost, US$ 46.3; 95% CI, 28.3‐64.4), and accommodation (mean annual cost, US$ 27.8; 95% CI, 4.4‐60.0) accounted for 16.7%, 2.2%, and 0.3% of total direct costs, respectively (Table 3). The overall mean indirect annual costs due to hypertension care seeking was US$ 171.7 (95% CI, 152.8‐190.5).

### 3.3 Impact on household income and coping strategies

**Figure 2:**
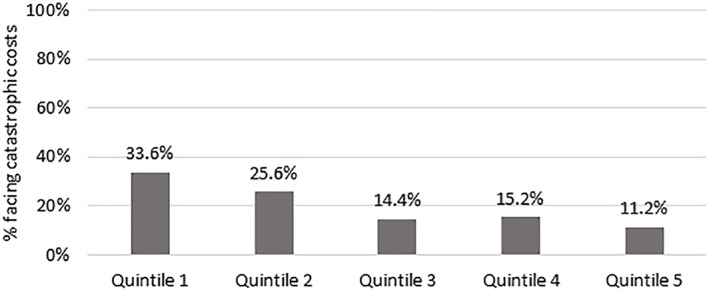
Relationship between catastrophic costs and socio‐economic status (Social‐economic status is represented by wealth quintiles: Quintile 1 represents the lowest socio‐economic group, while 5 represents the highest)

Costs for hypertension services were catastrophic for more than half (59%; 95% CI, 52.2‐65.4) of the households if all direct costs were considered. Alternatively, 43.3% (95% CI, 36.8‐50.2) of households experienced catastrophe if transport costs were excluded. Among respondents experiencing catastrophic costs, the poorest group of patients incurred higher direct costs with fewer resources to pay for it (Figure 2). The study found that most of the patients were unable to pay for hypertension treatment from their existing income sources and had to rely on savings (43.9%), borrowing from family/friends (25.1%), or sale of assets (31%), of which 33.3% was livestock. None of the hypertension patients reported receiving reimbursement for OOP costs incurred from either an insurance company or employer when seeking care for hypertension.

### 3.4 Productivity and social impact of hypertension

Patients were asked to estimate the number of work days they missed in the last 3 months due to hypertension. Of the 212 respondents, 30% reported they missed a median of 17 (IQR, 6‐46) working days. Forty‐two percent of respondents reported disrupted social life due to hypertension, affecting sexual life (n = 27), job loss (n = 46), divorce (n = 5), and separation from spouse (n = 10).

## 4 DISCUSSION

This study has documented patient costs for adults with hypertension that sought care in five public health care facilities in Kenya. Our findings show that patients cost for hypertension are driven by medicines. This is particularly worrisome given that unaffordability of hypertensive medicines is a likely cause of uncontrolled hypertension.^5^, ^22^, ^23^, ^33^, ^34^, ^35^, ^36^ Consequently, this is likely to interfere with continuity and comprehensiveness of care as patients only seek care when they have access to funds.^37^, ^38^ About half (57.7%) of the hypertensive patients reported they obtained their medicines from a public hospital. This suggests the possibility of infrequent antihypertensive drug supply in the sampled facilities. Medicines availability is a major factor influencing health care seeking behaviour. 39, 40, 41 Strategies to strengthen and expand access programmes to improve the availability of medicines for chronic diseases including antihypertensive medicines should be implemented to address such deficiencies.^42^, ^43^ Additionally, health being a devolved function in Kenya, county governments should develop mechanisms to ensure regular supply of antihypertensive medicines in public facilities and more generally improve financial access to these medicines.

Patients reported a median time of 30 minutes to access the clinics either by public means of transport or by walking; the cost of travelling to the medical clinics for different care seeking episodes was found to be a significant driver of patient costs. For instance, while the incidence of catastrophic health care costs was 43.3% (95% CI, 36.8‐50.2), it increased to 59.0% (95% CI, 52.2‐65.4) when transport costs were included. This could have negative access effects especially on poor patients as high transport costs and long distance to health facilities have been shown to hinder the poor from benefiting in pro‐poor health financing reforms in Kenya. 44

A high number of patients (36%) reported a sick visit out of their scheduled clinic appointment. This indicates that poor management of hypertension impacts both the health and the economic burden for patients as evidenced by high costs on transport (mean annual cost, US$ 173.9; 58.0‐289.7) and medication (mean annual cost, US$ 140.8; 91.7‐189.9). In addition, high user charges seen in all care seeking episodes, particularly during an inpatient admission, are potential barriers to care seeking given that the negative impacts of user charges in low resource settings, which have been well documented.^34^, ^37^, ^45^ There is substantial variation in patient costs between the inpatient and outpatient care seeking episodes. Inpatient admission costs represent a substantial economic burden.

Patients had to sell their property in order to meet associated costs of hypertension management in keeping with other assessment of the impact of health expenditures on households.^46^ The fact that about 36.1% of the Kenyan population live below the national poverty line^47^ indicates that patients are unable to meet treatment‐related expenditure for hypertension and had to adopt negative coping strategies such as borrowing or relying on family or social networks for assistance. One of the interventions for achieving the UHC goal of financial risk protection being pursued in Kenya and other LMICs is expansion of coverage under prepayment schemes such as social health insurance.^48^, ^49^ Our results show that three quarters of sampled hypertensive patients were not subscribed to any health insurance scheme reflecting coverage levels at a national level in Kenya where 80% of the population are not covered by any health insurance scheme.^17^, ^19^ This is similar to findings made by Kankeu et al^5^ in a review of financial burden of NCDs in LMICs. This means that hypertensive patients, especially those belonging to the lower rungs of the income ladder, bear a disproportionately higher burden of OOP, hence making them certain candidates to “medical poverty trap” where poor patients have to cope with the effect of reduced disposable income for other consumptions, which in turn increases poverty.^50^

High indirect costs (in terms of waiting time and the resultant productivity hours lost) were reported in this study during care seeking episodes. This is common in public health facilities and has been identified as a major disincentive of seeking care in public facilities among employed hypertensive patients in South Africa.^51^

### 4.1 Limitations

This study had several limitations. First, the study was not nationally representative as it was carried out in five health facilities in two rural counties. Secondly, the use of an official minimum wage to estimate productivity loss for all patients could have potentially overestimated indirect costs among patients who were unemployed prior to their illness or underestimated indirect costs among those who were employed.^30^ Recall bias is a concern in patient cost surveys. The study only focused on OOP costs associated with seeking care and does not include costs associated with those who did not seek care. These costs would be important in giving a comprehensive picture of OOP health care costs associated with hypertension care. These limitations notwithstanding, the data presented are potentially useful as inputs in costing and/or cost‐effectiveness models that require patient cost and suggest there are significant OOP costs associated with hypertension management in public facilities in Kenya, which offer a barrier to access to care.

## 5 CONCLUSION

The study demonstrates that hypertension places a considerable economic burden on patients in Kenya. As Kenya reforms its health system to prioritize the attainment of UHC, our results suggest that there is need for interventions to provide financial risk protection to individuals with a chronic disease such as hypertension. Given that medicines are a key cost driver for patient out‐of‐pocket costs, one approach would be to explicitly include hypertension medicines in the universal health care benefit package that Kenyan citizens are entitled to.

## ETHICS STATEMENTS

The scientific and ethics review unit at Kenya Medical Research Institute (KEMRI/SERU/CGMR‐C/041/3270) granted ethical approval for the study. County department of health officials and facility managers in respective counties provided permission to conduct the study in the selected facilities, and written informed consent was obtained from respondents.

## AVAILABILITY OF DATA AND MATERIAL

All the data for this study are available, and it can be accessed at the KEMRI‐Wellcome Trust Research Programme, subject to institutional data governance committee policies.

## AUTHOR CONTRIBUTIONS

Edwine Barasa, Anthony Etyang, Kenneth Munge, Jane Mbui, Zipporah Bukania, Fredrick Kirui, and Andrew Obala conceived the study. Robinson Oyando, Martin Njoroge, Antipa Sigilai, Kenneth Munge, Peter Nguhiu, and Edwine Barasa contributed to the development of data collection tools. Robinson Oyando collected the data. Robinson Oyando and Edwine Barasa analysed the data. Robinson Oyando developed the first draft of the manuscript. All authors contributed to writing subsequent versions of the manuscript
